# Recent Developments in Personal Glucose Meters as Point-of-Care Testing Devices (2020–2024)

**DOI:** 10.3390/bios14090419

**Published:** 2024-08-27

**Authors:** Dan-Ni Yang, Shan Geng, Rong Jing, Hao Zhang

**Affiliations:** 1 Chongqing Engineering Research Center of Pharmaceutical Sciences, Chongqing Medical and Pharmaceutical College, Chongqing 401331, China; 2220089@cqmpc.edu.cn (D.-N.Y.); girljr@163.com (R.J.); 2 The Central Laboratory, The Affiliated Dazu Hospital of Chongqing Medical University, Chongqing 402360, China; gengshan@cqmu.edu.cn

**Keywords:** personal glucose meters, non-glucose target, enzymatic transduction, nanocarrier transduction, glucose consumption transduction

## Abstract

Point-of-care testing (POCT) is a contemporary diagnostic approach characterized by its user-friendly nature, cost efficiency, environmental compatibility, and lack of reliance on professional experts. Therefore, it is widely used in clinical diagnosis and other analytical testing fields to meet the demand for rapid and convenient testing. The application of POCT technology not only improves testing efficiency, but also brings convenience and benefits to the healthcare industry. The personal glucose meter (PGM) is a highly successful commercial POCT tool that has been widely used not only for glucose analysis, but also for non-glucose target detection. In this review, the recent advances from 2020 to 2024 in non-glucose target analysis for PGMs as POCT devices are summarized. The signal transduction strategies for non-glucose target analysis based on PGMs, including enzymatic transduction, nanocarrier transduction (enzyme or glucose), and glucose consumption transduction are briefly introduced. Meanwhile, the applications of PGMs in non-glucose target analysis are outlined, encompassing biomedical, environmental, and food analysis, along with other diverse applications. Finally, the prospects of and obstacles to employing PGMs as POCT tools for non-glucose target analysis are discussed.

## 1. Introduction

Point-of-care testing (POCT) is an assay known for its rapidity, accuracy, simplicity, cost-effectiveness, and independence from specialized equipment, making it extensively applied in analytical testing. The current development of POCT has benefited from significant advances in biosensor technology, nanotechnology, and microfluidics. These innovations have enabled the miniaturization of testing devices, making them more portable and easier to use. The scope of the applications of POCT technology is expanding, providing more convenient and efficient testing in medical diagnostics, environmental monitoring, and food safety. Furthermore, the wireless transmission feature of POCT devices enables the direct transfer of test data to electronic health record systems, enhancing patient condition monitoring and outcome tracking. Consequently, some studies have investigated POCT products with wireless connectivity, such as pressure meters [1], thermometers [2], pH meters [3], personal glucose meters (PGMs) [4], smartphones [5], and electronic balances [6].

PGM technology has found widespread commercial use as a highly successful instant diagnostic tool. These portable devices allow patients to conveniently and accurately monitor their blood glucose levels at home, providing important support for effective diabetes management. This method demonstrates the capacity to detect glucose levels within the range of 0.6–33.0 mmol/L [7]. While PGM devices are effective for monitoring glucose levels, their utility is restricted when it comes to non-glucose targets. Hence, there is a necessity to develop glucometer-based assays for non-glucose target analysis. Lu’s group reported for the first time an innovative method for detecting other biomarkers with a portable PGM [8]. The detection principle of the method is as follows: a functional DNA-converting enzyme complex, which can specifically respond to the target analyte, is immobilized on magnetic beads (MBs). When the target analyte is present in the sample, the binding of the functional DNA interferes with DNA hybridization, causing the DNA invertase enzyme complex to detach from the MBs and be released into the solution. Upon removal of the MBs by an applied magnetic field, the DNA invertase enzyme complex demonstrates efficient catalysis of sucrose hydrolysis to glucose, allowing for quantitative detection using a PGM. The concentration of target analytes can be indirectly assessed using a PGM, as there is a quantitative relationship between the DNA invertase enzyme complexes released into the solution and the target analytes present in the sample. This technique has the ability to identify cocaine (detection limit of 3.4 μM), adenosine (18.0 μM), tuberculosis interferon (2.6 nM), and toxic uranium ions (9.1 nM) [8]. This study paved the way for extending the application of PGMs to identifying non-glucose targets. Concurrently, significant technological advancements in PGMs have broadened their capabilities in POCT diagnostics, enabling the detection of a variety of biologically relevant molecules beyond glucose.

**Figure 1:**
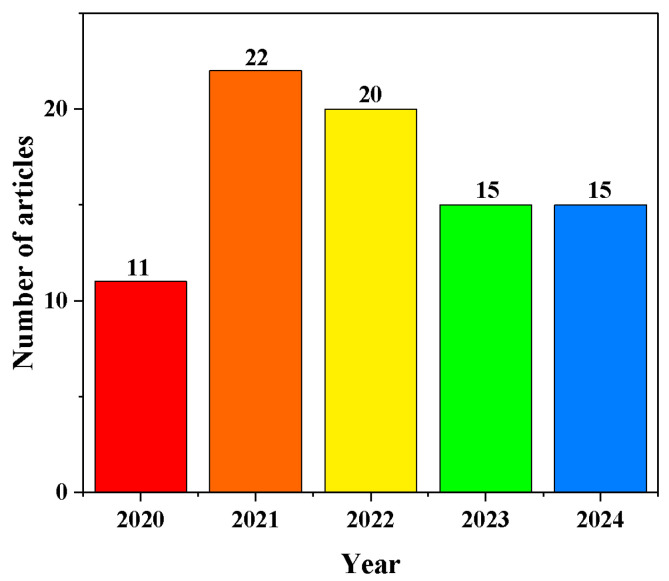
Number of published articles on PGM-based methods from 2020 to 2024. Data obtained from Web of Science. Search condition: Topic: personal glucose meter; Document types: Article; Total: 83 published articles up to August 2024.

**Figure 2:**
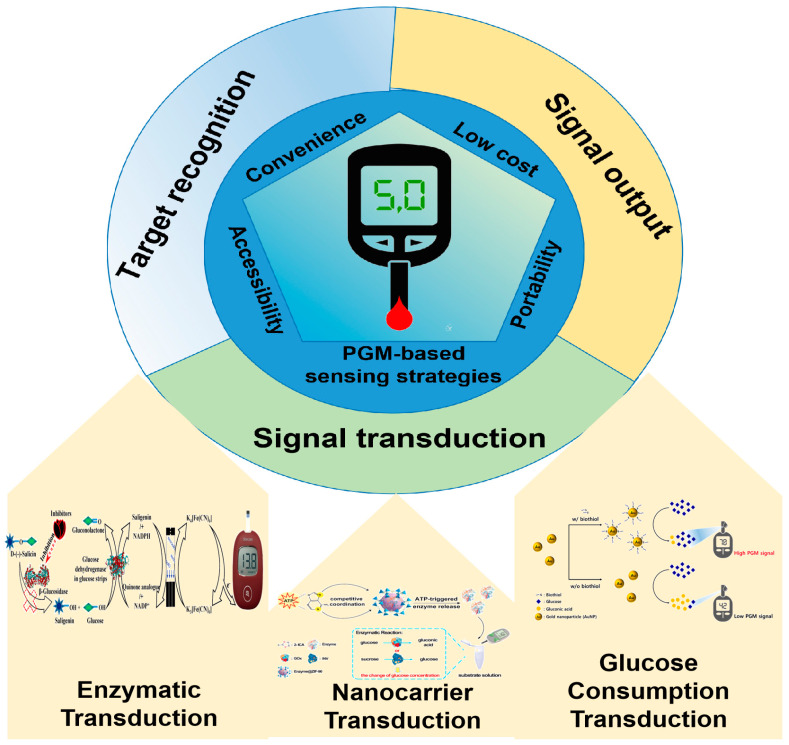
Visual overview of the review on PGM-based sensing strategies to detect analytes other than glucose. Reprinted with permission from Refs. [13,14,15].

There is a growing demand for the development of innovative PGM techniques to be used in analyzing non-glucose substances. To date, there have been several reviews on the use of PGMs for the analysis of non-glucose targets [9,10,11,12]. Some of these reviews present studies on the application of PGM technology in non-glucose target analysis up to 2020 [9,10], while others focus only on the application of PGM technology to food safety hazard analysis [11,12]. In contrast to these previous review papers, this comprehensive review outlines recent advancements in the field of PGM technology from 2020 to 2024, focusing on advances that make PGM devices versatile point-of-care tools for analyzing non-glucose targets (Figure 1). Moreover, this review not only covers food safety hazard analysis, but also highlights the versatility of PGM devices in analyzing various targets. Then, we explore the innovative signal transduction strategies (including enzymatic transduction, nanocarrier transduction, and glucose consumption transduction, especially in the application of nanozymes as non-glucose target recognition molecules) that support this expanded analytical capability and discuss how these developments can be applied in diverse areas such as biomedical analysis, food safety, and environmental monitoring (Figure 2). Finally, we also discuss how to fully utilize the advantages of these innovative signal transduction strategies to further enhance analytical capabilities and provide more possibilities for future research and applications.

## 2. Signal Transduction Strategies for Non-Glucose Target Analysis

In the detection process of glucose by PGM, the mechanism can be concisely outlined as follows: The glucose is transformed into reduced receptors through the catalytic action of glucose dehydrogenase or glucose oxidase. This reduction of the receptor on the glucose test strip initiates an electron transfer process. The PGM promptly detects this electron transfer, enabling precise measurement of glucose levels. However, the ability of a PGM to measure non-glucose target analytes depends on its innovative signal transduction strategy, which converts the presence of a target analyte into a measurable signal. The principle of the mechanism of PGMs for non-glucose target analytes involves three essential stages: target identification, signal conversion, and signal output. Of these steps, signal transduction is crucial and consists of three main approaches: enzymatic transduction, nanocarrier transduction, and glucose consumption transduction.

### 2.1. Enzymatic Transduction

Enzymatic transduction is a versatile and sensitive approach that has been extensively explored for analyzing non-glucose targets using PGMs. Enzymatic transduction relies on the utilization of an enzyme that exhibits specificity towards a non-glucose substrate. This enzyme catalyzes a reaction with the target analyte, resulting in the production of a product that is detectable by the PGM. The product may either directly interact with the existing glucose-sensing mechanism of the PGM or require additional mediators to generate a detectable signal. Enzymatic transduction offers a significant benefit in terms of specificity due to the ability to choose an enzyme with high affinity and selectivity for the target analyte. This selectivity minimizes interference from other substances in the sample, enhancing the accuracy of the results. Moreover, enzymatic transduction boasts high sensitivity and rapid detection capabilities, making it a commonly used technique in biomedical research and clinical diagnosis.

**Figure 3:**
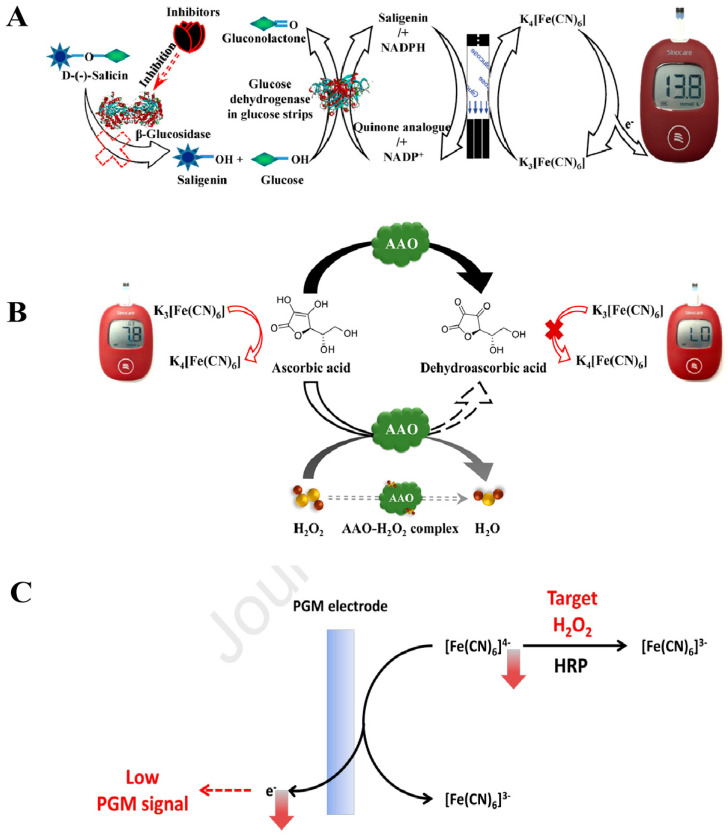
Schematic diagram of the enzymatic transduction technology for detecting analytes other than glucose using the PGM method. (**A**) Diagram illustration of the principle of the PGM method based on β-Glucosidase-mediated cascade enzymatic reaction. Reprinted with permission from Ref. [13]. (**B**) Diagram illustration of the design of H_2_O_2_ detection based on the inhibition of H_2_O_2_ on AAO and the PGM readout triggered by AA. Reprinted with permission from Ref. [17]. (**C**) Diagram illustration of the PGM strategy to detect H_2_O_2_ based on the target-induced oxidation of [Fe(CN)_6_]^4−^ to [Fe(CN)_6_]^3−^. Reprinted with permission from Ref. [18].

An easy-to-use technique employing PGM was established by us [13] for the evaluation of enzyme activity and the identification of inhibitors *via* a cascade reaction facilitated by β-glucosidase. As illustrated in Figure 3A, β-glucosidase facilitates the breakdown of D(-)-salicin into glucose and salicyl alcohol. The measurement of the generated glucose can be conducted using the PGM. Simultaneously, salicyl alcohol is capable of reducing ferricyanide to ferrocyanide in a redox reaction. Consequently, ferrocyanide undergoes re-oxidation in the glucose test strip, leading to the generation of an observable PGM signal. With the addition of a β-glucosidase inhibitor, the inhibitory effect causes a decrease in product yield and a reduction in the PGM signal. Finally, the quantification of β-glucosidase activity in bitter almond samples was carried out. The detection limit for β-glucosidase activity is 0.45 U/mL, with an activity range of 1.0–9.0 U/mL.

In addition to the glucose-sensing mechanism of the PGM mentioned above, the target analyte can also be detected in the glucose test strip through a direct chemical interaction with ferricyanide. For example, a PGM technique that employs an alkaline phosphatase (ALP)-mediated enzymatic reaction to measure ALP activity was developed by us [16]. The principle is as follows: The ALP facilitates the conversion of amifostine to ethanethiol (WR-1065) through hydrolysis, leading to the reduction of ferricyanide to ferrocyanide. This reduction reaction is followed by the oxidation of ferrocyanide on the glucose test strip, thereby yielding an observable PGM readout. If an ALP inhibitor is present, the activity of ALP is suppressed, leading to a lower amount of WR-1065 produced by the enzymatic reaction and a lower PGM readout. The detection limit for ALP activity is 0.13 U/μL, with an activity range of 0.33–3.33 U/μL.

Hydrogen peroxide (H_2_O_2_), classified as a reactive oxygen species, is formed as a byproduct of oxidative metabolism in organisms and serves as a key signaling molecule in multiple biological processes. Therefore, it is crucial to conduct quantitative detection on it. A rapid and portable method that relies on the ascorbate oxidase (AAO)-medicated reaction for the quantitative analysis of H_2_O_2_ was developed by us [17]. As shown in Figure 3B, ascorbic acid (AA), acting as a reducing agent, induces the conversion of ferricyanide to ferrocyanide on the glucose test strip, thereby producing an observable PGM signal. Simultaneously, AA undergoes catalysis by AAO to form dehydroascorbic acid. When H_2_O_2_ is introduced into the sample to form the H_2_O_2_–AAO complex, it inhibits the catalytic activity of AAO towards AA, leading to a rise in residual AA that is measured by PGM. Thereafter, a direct proportionality is present between the concentration of H_2_O_2_ and the residual AA concentration. The established method can detect H_2_O_2_ in the range of 2.5–5 × 10^3^ μM with a detection limit of 2.5 μM. Moreover, Lee et al. [18] introduced an innovative technique for detecting H_2_O_2_ under the catalysis of horseradish peroxidase (HRP). As depicted in Figure 3C, [Fe(CN)_6_]^4−^ undergoes oxidation to produce an observable PGM signal. H_2_O_2_ as an oxidant can be consumed to promote the oxidation process of ferrocyanide to ferricyanide under the catalysis of HRP. With the increase in H_2_O_2_ amount, the amount of ferrocyanide will decrease. Accordingly, the PGM signal readout will also decrease. Finally, the established approach can be used to detect H_2_O_2_ in the range of 0–40 μM with a detection limit of 3.63 μM. In addition, a series of enzyme-mediated reactions with PGM, such as with microRNAs [19], inorganic pyrophosphatase [20], lipase [21], tyrosinase [22], acetylcholinesterase [23], ALP [24], acarbose, and miglitol [25], have successfully been used to quantify non-glucose targets. However, there are still some challenges with enzymatic transduction, such as enzyme stability, cost, and potential interference from sample matrices, which must be addressed.

### 2.2. Nanocarrier Transduction

Nanomaterials offer a significant advantage as detection carriers owing to their expansive specific surface area, which allows for increased immobilization of target analytes and improved sensor sensitivity. Additionally, their excellent biocompatibility helps to maintain a stable environment and preserve the activity of biomolecules. As nanotechnology continues to progress, functional nanocomposites are increasingly utilized in POCT.

#### 2.2.1. Enzyme

The enzyme is first immobilized on the surface of the nanomaterial. When the target analyte triggers the release of the immobilized enzyme, the released enzyme is able to catalyze the generation of glucose from the substrate and bind to the PGM to achieve amplified detection of the non-glucose target analytes, thereby improving detection sensitivity. This method mainly consists of the following steps: (1) preparation of functionalized nanocarriers; (2) immobilization of the enzyme on the nanocarriers through physical, chemical, or biological interactions; (3) the release of the immobilized enzyme occurs when the target analytes specifically bind to the functionalized nanocarriers; and (4) the released enzyme catalyzes the transformation of the substrate into glucose, which is subsequently detected by the PGM.

**Figure 4:**
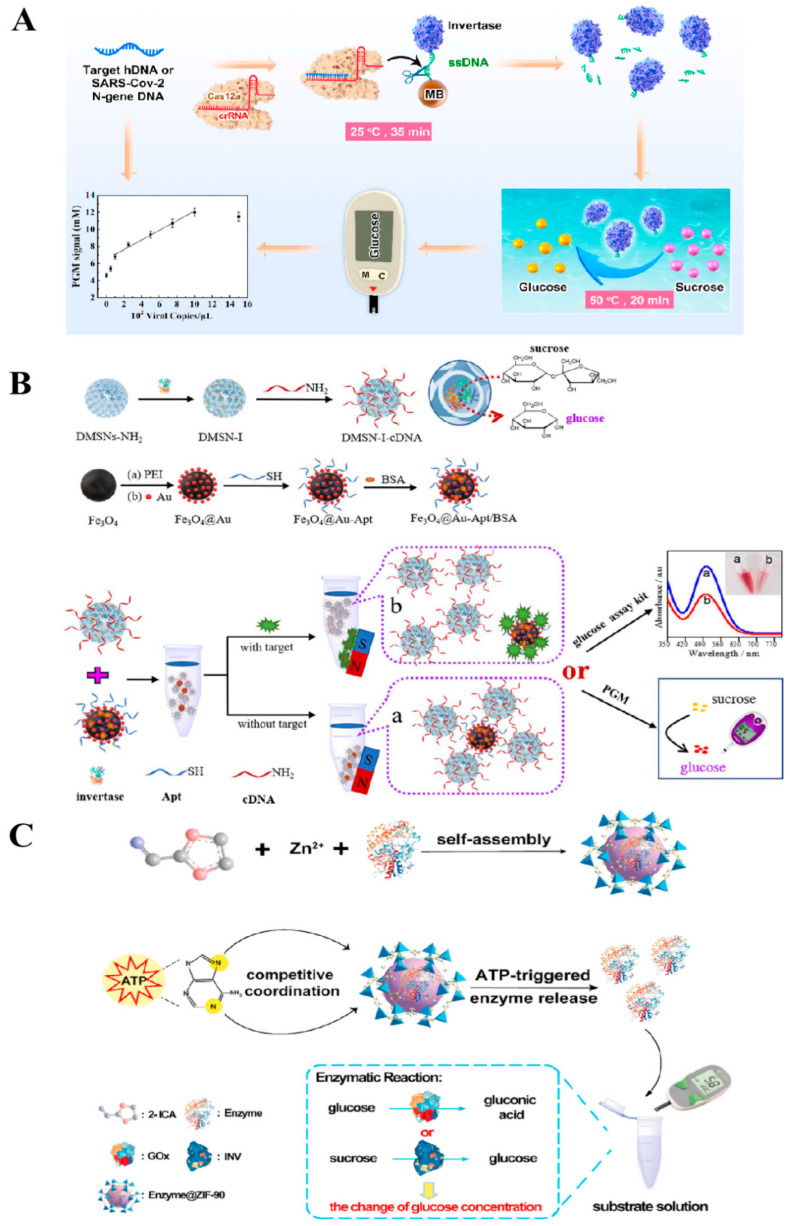
Schematic diagram of the nanocarrier-immobilized enzyme transduction technology for detecting analytes other than glucose using the PGM method. (**A**) Diagram illustration of the principle of a biosensing platform for pathogen DNA based on the CRISPR Cas12a system and PGM method. Reprinted with permission from Ref. [26]. (**B**) Diagram illustration of the principle of the Fe_3_O_4_@Au-Apt/DMSNs-I-cDNA aptamer sensor for the detection of aflatoxin B1 and bisphenol A by PGM. Reprinted with permission from Ref. [27]. (**C**) Diagram illustration of the principle of the enzyme@ZIF-90 platform for ATP detection by PGM. Reprinted with permission from Ref. [14].

Fang et al. [26] combined a clustered regularly interspaced short palindromic repeat assay for Cas12a with a PGM detection technique to achieve the detection of pathogenic nucleic acids. As shown in Figure 4A, the first step in the procedure involved immobilizing an ssDNA invertase coupler onto the surfaces of MBs through a biotin–streptavidin interaction. Following this, the pathogen’s target DNA bound to Cas12a-crRNA and became activated and cleaved by the single-stranded DNA, resulting in the liberation of invertase from the MBs. After magnetic separation was conducted with an applied magnetic field, the supernatant was subjected to incubation with the substrate sucrose at 50 °C, followed by assessment using the PGM method. This method enables the ultrasensitive analysis of HIV-associated DNA in human serum samples or SARS-CoV-2 virus in saliva samples with detection limits of 11.0 fM and 50 copies/μL, respectively.

To increase the enzyme loading levels and ensure the sustained catalytic efficiency of the immobilized enzyme, Yang et al. [27] employed dendritic mesoporous silica nanoparticles (DMSNs) possessing a substantial specific surface area to immobilize invertase and develop a PGM-based aptamer sensor, which was able to achieve high sensitivity for detecting aflatoxin B1 (AFB1) and bisphenol A (BPA) in corn oil and wheat flour. Figure 4B illustrates the immobilization process of the invertase within the mesopores of silica nanoparticles using a cross-linker. Subsequently, the complementary DNA strand of the aptamer (cDNA) was engineered onto the DMSNs invertase supports (DMSNs-I). The thiol-terminal aptamer (SH-Apt) was then anchored onto the Fe_3_O_4_@Au nanoparticles to produce the Fe_3_O_4_@Au-Apt capture probe. Upon co-incubation of Fe_3_O_4_@Au-Apt/DMSNs-I-cDNA with the target analyte, the DMSNs-I-cDNA was released as a result of the selective binding of Fe_3_O_4_@Au-Apt to the target analyte. Then, the liberated DMSNs-I-cDNA facilitated the transformation of sucrose into glucose, which was detectable by PGM. This process establishes a direct correlation between the PGM signals and the concentration of the target analyte. This study employed DMSNs to encapsulate enzymes, resulting in a substantial increase in enzyme loading (407.3 ± 28 mg/g) while retaining 78.56% of their biological activity. The AFB1 labeling approach introduced in this study yielded an approximately 3.19-fold improvement in sensitivity and a two-order-of-magnitude decrease in the detection limit compared to sensors labeled with a single enzyme. The method demonstrated detection limits of 0.74 pg/mL for AFB1 and 0.094 pM for BPA, with recovery rates ranging from 97.72% to 103.84% for AFB1 and from 99.46% to 110.33% for BPA in real sample analysis.

Although signal transduction PGM technology based on enzyme-immobilized nanocarriers has been successfully applied in non-glucose target detection, there are still some obvious shortcomings. These include complex enzyme coupling processes, potential impacts on the structure and function of enzymes due to immobilization, and cumbersome overall operation. These limitations require further research and improvement. To meet the growing demand for non-labeled target detection, Cao et al. [14] utilized the excellent enzyme encapsulation ability and stimulus responsiveness of metal–organic frameworks to successfully embed glucose oxidase or invertase in ZIF-90, which allowed for detecting adenosine triphosphate (ATP) in serum samples by PGM analysis. As shown in Figure 4C, Enzyme@ZIF-90 detection probes (Enzyme@ZIF-90) were prepared using a one-pot method. When the target analyte ATP is incubated with Enzyme@ZIF-90, the bases in ATP will compete for coordination with the ZIF-90 binding sites, causing the collapse of the ZIF-90 structure and releasing either glucose oxidase or invertase. The released enzymes can catalyze either the oxidation of glucose or the hydrolysis of sucrose, resulting in an alteration in the glucose concentration that can be identified using PGM. Finally, this method was utilized to quantify ATP in human serum, demonstrating a detection limit of 233 nM and a recovery rate within the range of 96.01% to 103.24%.

#### 2.2.2. Glucose

**Figure 5:**
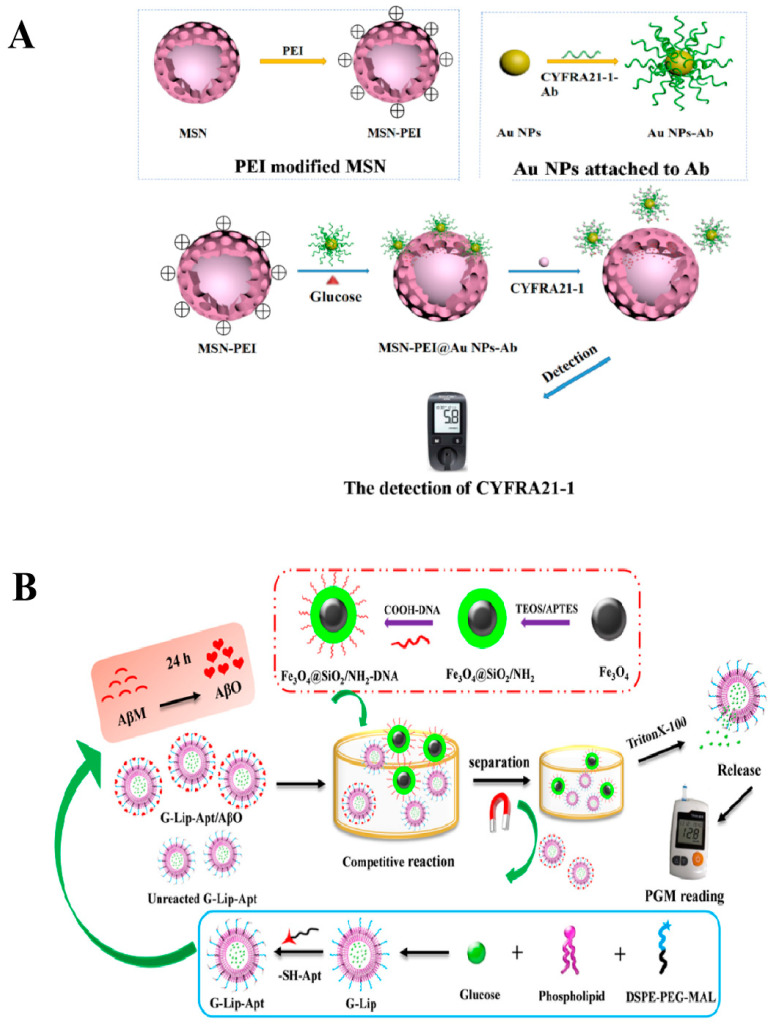
Schematic diagram of nanocarrier-encapsulated glucose transduction technology for detecting analytes other than glucose using the PGM method. (**A**) Diagram illustration of the principle of a PEM-based MSN-PEI@Au NPs-Ab platform for CYFRA21-1 detection. Reprinted with permission from Ref. [28]. (**B**) Diagram illustration of the preparation of aptamer-grafted liposomes with glucose encapsulation (G-Lip-Apt), single-strand DNA-attached magnetic Fe_3_O_4_@SiO_2_/NH_2_-DNA nanocomposites, and the principle of AβO detection by PGM. Reprinted with permission from Ref. [30].

In contrast to techniques that encapsulate or immobilize enzymes on nanocarriers, encapsulating glucose directly in nanomaterials and then releasing it in the presence of a specific target analyte to be detected by a PGM is a very promising strategy. This approach typically comprises two distinct stages: the initial encapsulation of glucose molecules within mesoporous materials through physical or chemical methods, followed by the disruption of the encapsulation layer of the mesoporous material by the target analyte, leading to the release of glucose for detection using PGM. Mesoporous nanomaterials are commonly used to encapsulate a variety of molecules, such as drugs, dyes, and proteins, due to their ability to adjust pore size uniformly. The material shows significant promise in the field of controlled release. Lv et al. [28] employed negatively charged CYFRA21-1 antibody-conjugated gold nanoparticles (Au NPs-Ab) to entrap glucose molecules within the pores of positively charged polyethyleneimine-modified mesoporous silica nanoparticles (MSNs-PEI). In the presence of the CYFRA21-1 antigen, as depicted in Figure 5A, the specific binding to Au-NPs-Ab triggers the release of encapsulated glucose. The quantity of glucose released correlates directly with the concentration of the CYFRA21-1 antigen, thereby establishing a linear relationship between CYFRA21-1 antigen levels and the PGM signal. Finally, this method facilitates the analysis of CYFRA21-1 across a range of 1.3 ng/mL to 160 ng/mL in human serum, boasting a remarkably low detection limit of 0.79 ng/mL. A similar study was also reported by Li et al. [29] to detect the environmental pollutant of *p*-aminophenol (PAP) based on the PGM method with MSN@MnO_2_ nanoprobes. The pores of MSN nanocarriers were initially loaded with glucose. Subsequently, MnO_2_ nanosheets were incorporated onto the Glu-MSNs’ surfaces using an in situ synthesis approach to prevent glucose leakage. When PAP was present in the sample, MnO_2_ would undergo a redox reaction with PAP, destroying the MnO_2_ wrapping layer and triggering the release of glucose. The concentration of glucose in the solution can be measured by PGM, which can indirectly reflect the amount of PAP in the sample. Finally, this strategy achieved a detection limit of 2.78 μM for PAP with a linear range of 10.0–400 μM and a recovery rate of 96.7–101.0%.

In addition to MSNs, which are often used for encapsulating glucose, liposomes with a spherical bilayer structure have also been used as carriers for encapsulating glucose molecules. This liposome encapsulation system helps to amplify the relevant biological signals for detection. For instance, Hu et al. [30] developed a glucose-based liposomal portable biosensor using PGM aptamers to detect Aβ oligomers (AβO). The sensor consisted of aptamer-grafted liposomes encapsulating glucose (G-Lip-Apt) and single-stranded DNA (ssDNA)-modified magnetic nanocomposites (Fe_3_O_4_@SiO_2_/NH_2_-DNA), as shown in Figure 5B. In the presence of AβO, the G-Lip-Apt/AβO complex is formed because the sulfhydryl ligand on the G-Lip-Apt can be specifically recognized by AβO. Fe_3_O_4_@SiO_2_/NH_2_-DNA is then added to form a complex with the unbound G-Lip-Apt through complementary pairing. This complex can be separated using an applied magnetic field. When the surfactant Triton X-100 was added, glucose was released from the complex and detected by PGM. Finally, the method was utilized to detect Aβ oligomers, with a linear range of 5.0–1000 nM and a detection limit of 2.27 nM.

### 2.3. Glucose Consumption Transduction

**Figure 6:**
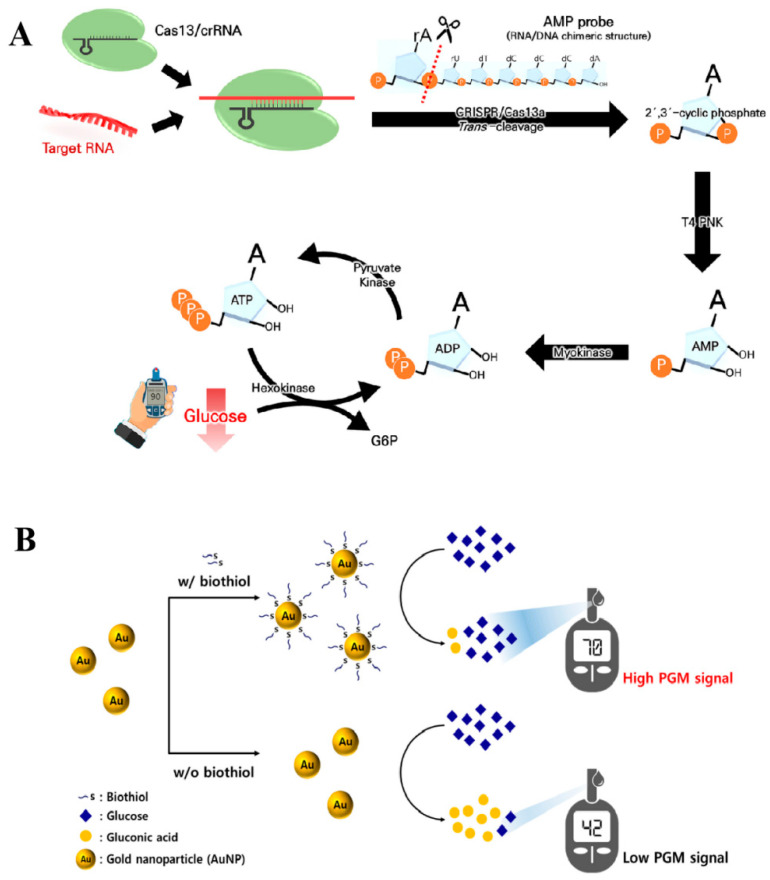
Schematic diagram of the glucose consumption transduction technology for detecting analytes other than glucose using the PGM method. (**A**) Diagram illustration of PGM-CeO_2_ NP-based TdT detection. Reprinted with permission from Ref. [31]. (**B**) Diagram illustration of PGM-AuNP-based biothiol detection. Reprinted with permission from Ref. [15].

The glucose consumption transduction strategy results in a decrease in the amount of glucose, mainly through direct or indirect consumption of glucose by the target analyte. Subsequently, the change in glucose level can be measured by PGM. For example, Park et al. [31] utilized an enzymatic cascade enzyme reaction (CER), which promotes the repeated consumption and production of adenosine diphosphate (ADP) through the association of hexokinase and pyruvate kinase, causing a continuous depletion of glucose (Figure 6A). When the RNA analyte is present, the *trans*-cleavage function of CRISPR/Cas13a becomes active, leading to the generation of 2′,3′-cyclic adenosine phosphate. Subsequently, T4 polynucleotide kinase enzymatically converts it to adenosine monophosphate (AMP). AMP is phosphorylated by myokinase to form ADP, which serves as a substrate in the enzymatic cascade reaction with pyruvate kinase and hexokinase. The entire process involves a stepwise transformation of glucose into glucose-6-phosphate, leading to a glucose level directly correlated with the target RNA quantity. This enables the indirect quantification of the RNA using the PGM. Finally, the method detected SARS-CoV-2 RNA up to 27 pM.

With extensive research on nanozymes having been conducted in the field of sensing technology, several nanozymes have been identified as possessing catalytic properties comparable to those of glucose oxidase. These nanozymes can modify their configuration in response to the target analyte, thereby affecting their catalytic efficiency. This modification results in a change in glucose levels, which are subsequently detected by the PGM. For example, Kim et al. [32,33] developed cerium oxide nanoparticles (CeO_2_) that demonstrate glucose oxidase-like behavior. This behavior catalyzes the oxidative decomposition of glucose and is detected by PGM. The aggregation of CeO_2_ nanoparticles in the presence of a target analyte results in diminished glucose oxidase-like catalytic activity. Conversely, in the absence of the target analyte, the CeO_2_ nanoparticles maintained a well-dispersed state in the solution, effectively promoting the oxidative decomposition of glucose into gluconic acid and causing a substantial decrease in the concentration of glucose. The method was effectively utilized for detecting terminal transferase in human blood and DNA in *Escherichia coli*, with detection limits of 0.7 U/mL and 10 copies of target gDNA, respectively. Moreover, a similar study has been reported by Lee et al. [15] for detecting biothiols by AuNPs mimicking glucose oxidase activity. As shown in Figure 6B, biothiols were able to interact with AuNPs and inhibit their glucose oxidase-like catalytic activity. Finally, this method enables the identification of cysteine, homocysteine, and glutathione with detection limits of 0.116 μM, 0.059 μM, and 0.133 μM, respectively.

## 3. Application of PGM in Non-Glucose Target Analysis

The applications of PGMs in various fields are vast and diverse. In the biomedical field, PGMs are used for disease biomarker detection. In food analysis, PGMs are utilized for detecting contaminants, monitoring food quality, and food safety. In environmental analysis, PGMs play a crucial role in detecting heavy metal pollutants. Furthermore, the versatility and distinct properties of PGMs make them indispensable in a diverse array of applications, including catalysis and drug screening. This section summarizes the diverse applications of PGMs in biomedical, food, environmental analysis, and other fields.

### 3.1. Biomedical Analysis

POCT technology is becoming increasingly essential in biomedical analysis, especially in the detection and prevention of clinical diseases. This is largely due to its cost-effectiveness, user-friendly operation, and non-invasive or minimally invasive characteristics. Among the various POCT tools available, PGMs stand out as one of the most successful commercialized products, widely used for the detection and analysis of biomarkers. Table 1 demonstrates the application of the PGM technique in biomedical research.

**Figure 7:**
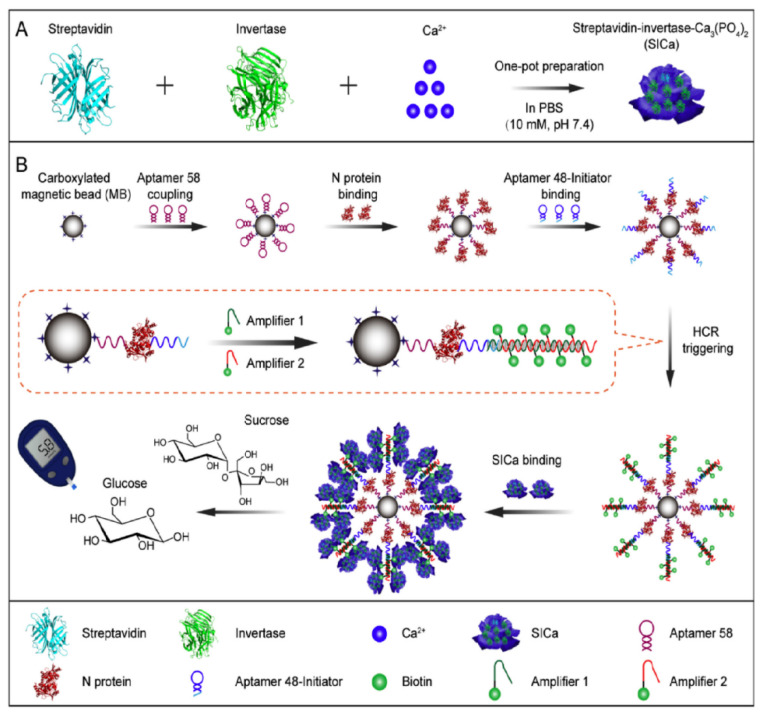
Application of PGM in non-glucose target quantitative biomedical analysis. (**A**) Preparation of streptavidin-invertase-Ca_3_(PO_4_)_2_ hybrid nanoflowers by one-pot method. (**B**) Overview of the proposed SICa-based N protein detection method. Reprinted with permission from Ref. [75].

The emergence of the novel coronavirus disease 2019 (COVID-19), caused by the severe acute respiratory syndrome coronavirus 2 (SARS-CoV-2), poses a significant threat to public health. Therefore, early prevention and detection of SARS-CoV-2 is of the utmost importance. Yin et al. [75] first synthesized a hybrid nanoflower complex (SICa) comprising streptavidin, invertase, and Ca_3_(PO_4_)_2_ through a one-pot method (Figure 7). Then, they immobilized aptamer 58, which was amino-functionalized, onto carboxylated MBs. After that, aptamer 58 can specifically identify the N protein of SARS-CoV-2, while the N protein captured by the MBs can non-competitively capture aptamer 48 and trigger a hybridization chain reaction. The two 5′ biotinylated DNA strands with cohesive ends are attached to the MBs through complementary pairing. The SICa and biotin-modified DNA conjugate to form a complex through streptavidin–biotin affinity. When sucrose is added to the complex, the invertase that was released facilitated the breakdown of sucrose into glucose, which is correlated with the amount of the SARS-CoV-2 N protein. Finally, this method exhibits a detection limit of 1 pg/mL for SARS-CoV-2 and achieves a recovery rate ranging from 93.50% to 107.30% for the N protein in both plasma and saliva.

The regulation of gene expression in cells is significantly influenced by miRNAs. Research has established a direct link between aberrant miRNA expression and the development of distinct diseases. For example, overexpression of miRNA-21 may be associated with cancer, cardiovascular disease, and kidney disease. Huang et al. [76] employed streptavidin and biotin interactions to immobilize biotinylated DNA invertase on streptavidin-coated MBs, resulting in the formation of an MB complex. The target miRNA-21 was captured by the MB complex through DNA/RNA hybridization. Subsequently, a double-stranded specific nuclease specifically cleaved the DNA, releasing the invertase from the MBs. The invertase that was released facilitated the breakdown of sucrose into glucose, which was later analyzed by PGM. Using this method, the detection limit for miRNA-21 can reach 1.8 pM, with a linear range of 10–200 pM. The recovery rate for miRNA-21 in urine samples is 94.7–107.0%.

The quantification of immunoglobulin E (IgE) holds significant clinical relevance for assessing immune function and facilitating the identification of immunoproliferative, immunodeficiency, and autoimmune disorders. Han et al. [77] utilized a CER to induce glucose consumption through the combination of hexokinase and pyruvate kinase, promoting repeated ATP consumption and production. When the target IgE is absent, ALP-modified secondary antibodies are eluted and separated under an applied magnetic field. ATP is converted to ADP by hexokinase, while glucose is converted to glucose-6-phosphate. Meanwhile, phosphoenolpyruvic acid and ADP are converted to pyruvate and ATP by pyruvate kinase, entering the next round of the CER and leading to the continuous consumption of glucose. Conversely, in the presence of the target IgE, the IgE attaches to the ALP-modified secondary antibody to form a sandwich complex. The initial ATP is hydrolyzed by ALP, resulting in a blockage of the CER reaction and an inability to consume glucose. The level of target IgE is positively correlated with glucose levels, with the detection of IgE up to 29.6 ng/mL and its recovery rate in plasma ranging from 99% to 105%.

### 3.2. Food Analysis

Recent years have seen a significant increase in the frequency of food safety incidents, which pose a notable risk to public health. Challenges such as microbiological contamination and pesticide residues have severely compromised the safety and quality of food. Consequently, the adoption of sophisticated analytical methods has emerged as a pivotal strategy to enhance food safety. Table 2 demonstrates the application of the PGM method in food analysis.

**Figure 8:**
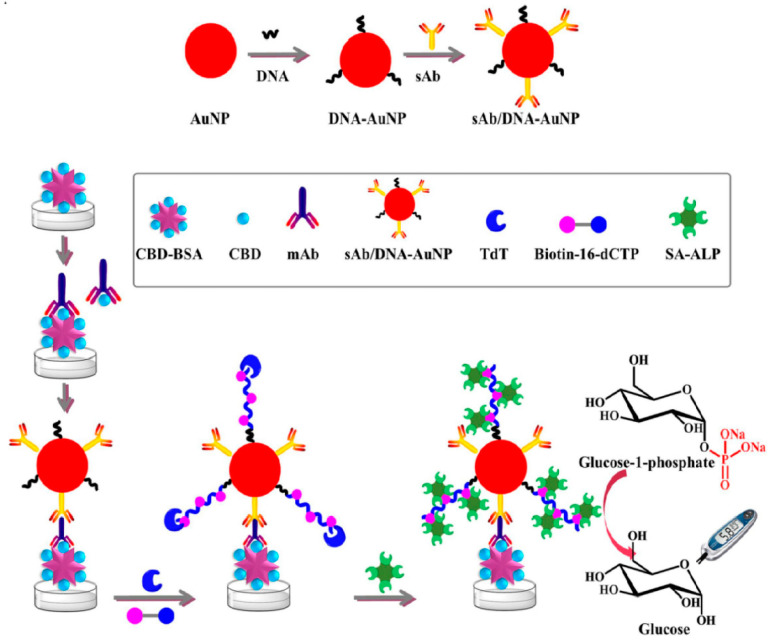
Applications of PGMs in non-glucose target quantitative food analysis. Schematic of the mechanism of the proposed portable ELISA for the detection of CBD. Reprinted with permission from Ref. [90].

Chlorothalonil (CBD) is frequently used in agriculture as a broad-spectrum fungicide to combat a diverse array of fungal diseases. Nevertheless, the introduction of pesticide residues into the human body has the potential to harm the endocrine system and interfere with hormone balance. Therefore, quantitative control is very important for CBD. Liu et al. [90] first attached DNA and secondary antibodies (sAbs) to AuNPs to form sAb/DNA-AuNP probes (Figure 8). When the target CBD is absent, the CBD monoclonal antibody (mAb) binds to the microplate coated with CBD–bovine serum albumin (BSA), forming a CBD-BSA-mAb complex. Through the interaction between the mAb and the sAb, the sAb/DNA-AuNP probe is then immobilized on the microplate. Next, terminal deoxynucleotidyl transferase and biotin-16-dCTP are added, allowing for the extension of single-stranded DNA on AuNPs and the introduction of multiple biotin binding sites. Subsequently, ALP functionalized with streptavidin is anchored onto the AuNPs through the specific affinity between biotin and streptavidin. On the other hand, ALP can catalyze the conversion of glucose-1-phosphate into glucose, which can then be analyzed by PGM. When CBD is present, it competitively binds to the mAb with CBD-BSA on the microplate, leading to a reduction in mAb binding sites and ultimately affecting glucose production. The method achieved a detection limit of 0.37 ng/mL and a linear range of 0.13–100 ng/mL for CBD. The recovery rates of CBD in canned citrus, citrus fruit, and cabbage samples were 70.4–109.4%.

Ochratoxins, including ochratoxin A (OTA), ochratoxin B, and ochratoxin C, are a class of toxic metabolites primarily synthesized by *Aspergillus* or *Penicillium strains* and commonly found in moldy cereals. These toxins pose significant health risks to humans, potentially causing liver and kidney damage, as well as compromising the immune system. Therefore, measures must be implemented during food production and storage to mitigate ochratoxin production and accumulation, thereby enhancing food safety. Zhang et al. [91] first hybridized the OTA aptamer and biotinylated signaling probe to form a double-stranded complex. Subsequently, the double-stranded complex was covalently attached to a carbon electrode modified with AuNPs through an Au-S interaction. Biotinylated invertase can be anchored onto the surface of the sensor through specific recognition. As the amount of OTA in the sample increases, it competes with the aptamer for binding, leading to the destruction of the formed dsDNA and a corresponding reduction in the amount of glucose. The limit of detection for OTA could reach 0.45 ng/mL with a linear range of 0.5–10 ng/mL, and the recovery rates of OTA in rice ranged from 88.0–103.0%.

### 3.3. Environmental Analysis

The issue of environmental pollution is a major hindrance to the progress of green development. Due to increasing human activity and rapid industrial growth, significant amounts of pollutants, particularly metal ions, are being discharged directly into the environment, specifically polluting water bodies. As a result, scientific research is increasingly focusing on the prompt and precise analysis of these environmental pollutants. The applications of the PGM method in environmental analysis are shown in Table 2.

**Figure 9:**
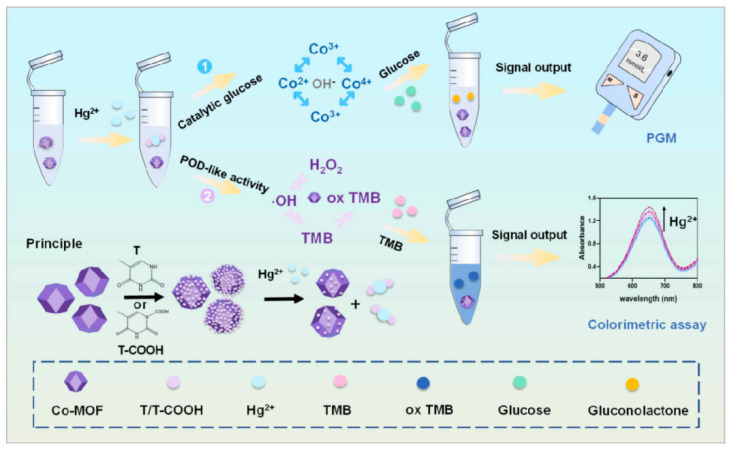
Applications of PGMs in non-glucose target quantitative environmental analysis. Schematic of the mechanism of the proposed two-mode Hg^2+^ sensing platforms based on the tunable cobalt metal–organic framework (Co-MOF) active site strategy for the detection of Hg^2+^. Reprinted with permission from Ref. [84].

The contamination of heavy metal ions presents a significant risk to both ecosystems and human health. Xu et al. [84] modified thymine (T) and thymine-1-acetic acid (T-COOH) onto the surface of cobalt metal–organic frameworks (Co-MOF), respectively, to form two complexes: Co-MOF@T and Co-MOF@T-COOH (Figure 9). However, the presence of T/T-COOH has the potential to obscure the active site of Co-MOF. When Hg^2+^ is present, the detachment of T/T-COOH from Co-MOF exposes its active site. Under alkaline conditions, Co-MOF exhibits glucose oxidase-like behavior, facilitating alterations in glucose levels and converting it into the PGM readout signal. The PGM method enabled the detection of Hg^2+^ in water at a concentration of 3.69 nM, with a linear range of 5–30 nM. The recovery rates for detecting Hg^2+^ in real samples ranged from 92.34% to 110.28%. Copper ions play a crucial role as essential trace elements in various biological metabolic processes within living organisms. In organisms, growth and metabolic disorders can arise from a copper deficiency, while an excess of copper can result in toxicity. Gu et al. [85] used glutaraldehyde cross-linking to immobilize streptavidin onto SiO_2_-modified Fe_3_O_4_ magnetic nanoparticles. Then, Cu-sub-invertase and Cu-Enz were also immobilized onto the magnetic nanoparticles through specific interactions between streptavidin and biotin, as well as hybridization reactions. When the target Cu^2+^ was present, it was able to cleave the DNA-Cu-sub and detach the invertase from the magnetic nanoparticles. The invertase enzyme facilitates the breakdown of sucrose into glucose, which is then identified using the PGM. The detection limit of Cu^2+^ in tap water reached 10 nM with a linear range of 0.01–5 μM, and its recovery rate ranged from 97.0% to 116.0%.

### 3.4. Other Applications of PGM

Using PGMs in non-glucose target analysis has demonstrated their utility in a range of fields beyond biomedical, food, and environmental analysis. For instance, PGMs as platforms for enzyme activity assays and high-throughput screening of enzyme inhibitors have been successfully developed by Tian et al. [92], the breakdown of maltose into glucose is catalyzed by α-glucosidase, with the resulting glucose levels quantifiable using the PGM method. When the α-glucosidase inhibitor is present, its catalytic activity decreases, resulting in a decrease in PGM readout. Therefore, screening for α-glucosidase inhibitors can be achieved without any complicated procedures. Finally, this strategy was employed to assess the inhibitory effects of 34 small molecule compounds and 18 plant extracts on α-glucosidase. As a result, protocatechualdehyde, lithospermic acid, 2,3,5,4′-tetrahydroxy stilbene-2-O-β-D-glucoside, and lemon extract were identified as having strong enzyme inhibitory activity. On the other hand, PGM-use can not only serve as a drug screening platform, but also for monitoring therapeutic drugs. Zhang et al. [25] introduced a simple PGM approach for monitoring the antidiabetic drugs acarbose and miglitol. The method is based on the enzymatic activity of α-glucosidase, which catalyzes the hydrolysis of 2-O-α-D-glucopyranosyl-L-ascorbic acid to generate ascorbic acid and glucose. The incubation of antidiabetic drugs with the enzyme resulted in a loss of enzyme activity, leading to a decrease in the quantity of enzymatic products. Finally, the PGM method enabled the identification of acarbose and miglitol at detection limits of 0.33 μM and 1.0 μM, respectively, with linear ranges of 1.0–30.0 μM and 3.0–33.3 μM. The recovery rates for acarbose and migliol in human serum samples are 89.6–114.5% and 93.9–106.5%, respectively. Gong et al. [93] developed a sensitive and cost-effective PGM approach for monitoring the site-specific cleavage efficiency of CRISPR-Cas9. The PGM method enabled the analysis of DNA cleavage of CRISPR-Cas9 at detection limits of 1.1 nM.

## 4. Conclusions and Perspectives

In conclusion, the recent focus has been on repurposing the PGM, a successful medical diagnostic tool, for the analysis of analytes other than glucose. The prospects of such innovations are considerable, as their effective utilization could potentially revolutionize the field of cost-efficient, portable analytical technology, allowing for POCT diagnosis of a broad spectrum of diseases. This review describes the PGM with common signal transductions and signal output strategies for non-glucose target analysis in different areas.

Despite the remarkable progress made between 2020 and 2024, PGMs also have some disadvantages: (1) Despite the versatility provided by enzymatic and nanocarrier transduction methods, challenges persist in achieving high selectivity and sensitivity with PGMs. Addressing these concerns necessitates additional research and enhancements (such as CER and CRISPR-Cas methods) to ensure the precision and reliability of PGM assays; (2) The high specificity of PGM for glucose limits its specificity for the detection of non-glucose substances. The presence of some structural analogs may lead to erroneous results, and current assay tools may not be sufficient to detect trace analytes; (3) Considering the variety of disease markers and the possibility of food and environmental contamination with multiple substances, it is imperative to broaden the scope of PGM assay targets from individual analytes to intricate multi-component systems; (4) Environmental factors, including temperature and humidity, have the potential to influence the stability of enzymes and nanocarriers, which in turn may reduce the reliability of measurement results; (5) Despite the user-friendly design of the PGM, the complexity of certain non-glucose testing functions necessitates specialized knowledge, potentially resulting in user errors that may impact the accuracy of test results. Therefore, the utilization of PGMs to detect non-glucose targets is still far from being commercially accessible.

Additional future research and development of PGMs for non-glucose analytes is needed to achieve the following goals: (1) Improvements in enzyme specificity and nanocarrier design are expected to increase the sensitivity and selectivity of PGMs for non-glucose targets; (2) Enhancing the capacity to detect multiple target analytes simultaneously through the development of integrated detection techniques, thereby optimizing analytical efficiency and cost-effectiveness; (3) Test strips specifically designed to detect non-glucose targets have made PGMs more compact and easier to use, making them available to a wider range of users and suitable for a variety of environments, including remote or resource-limited areas; (4) Future studies should aim to broaden the understanding of signal transduction pathways and innovate mechanisms for sensor recognition; (5) The application areas for PGMs need to be further broadened. For example, there could be applications for monitoring blood drug concentrations.

## Figures and Tables

**Table 1:** Application of PGMs in non-glucose target quantitative biomedical analysis.

Analytes/Real Sample	Signal Transduction Strategies	Limit of Detection	Linear Range	Ref.
MiRNA155/Human serum	Enzymatic transduction	0.36 fM	1 fM–10 nM	[34]
*Pseudomonas aeruginosa*/Medical apparatus	Enzymatic transduction	36 cfu/mL	100–1.00 × 10^7^ cfu/mL	[35]
Exosomes	Nanocarrier transduction	/	10^3^−10^6^ particles/μL	[36]
MiRNA-21/Commercial serum samples	Nanocarrier transduction	2.32 pM	10 pM–100 nM	[37]
Carcinoembryonic antigen/Serum sample	Nanocarrier transduction	0.28 ng/mL	2–200 ng/mL	[38]
Troponin I/Human serum sample	Nanocarrier transduction	0.001 ng/mL	0.002–250 ng/mL	[39]
DNA adenine methyltransferase/Human serum	Nanocarrier transduction	0.001 U/mL	0.001–5.0 U/mL	[40]
SARS-CoV-2 N-gene and PCB77/Human serum and water samples	Nanocarrier transduction	N-gene: 2.6 fM PCB77: 3.2 × 10^−5^ μg/L	N-gene: 10 fM–1.0 nM PCB77: 1.0 × 10^−4^–1.0 μg/L	[41]
Prostate cancer antigen 3/Urine	Nanocarrier transduction	/	5–100 pM	[42]
MiRNA-21/Urine of mice	Nanocarrier transduction	68.08 fM	100 fM–1 pM	[43]
MiRNA21 and miRNA205/Human serum	Nanocarrier transduction	miRNA21: 2.4 pM miRNA205: 1.1 pM	miRNA21: 10 pM–100 nM	[44]
Alpha-fetoprotein/Serum samples	Nanocarrier transduction	10 ng/mL	0.01–100 μg/mL	[45]
SARS-CoV-2 RNA/Human serum, plasma, and saliva	Glucose consumption	27 pM	0–1.28 nM	[31]
Pheochromocytoma 12 cell/peripheral circulating blood	Glucose consumption	3 cells/mL	4–10^5^ cells/mL	[46]
Nucleocapsid protein gene of SARS-CoV-2	Nanocarrier transduction	0.15 pM	/	[47]
Human papillomavirus 16 E6/E7 mRNA/Cervical swab samples	Nanocarrier transduction	5 fM	5 fM–10 pM	[48]
*mecA* gene in Methicillin-resistant *Staphylococcus aureus*/Serum from neonatal infection patients	Nanocarrier transduction	2 CFU/mL	10–1 × 10^4^ CFU/mL	[49]
Horseradish latent virus cloned DNA plasmids/plant lysates	Enzymatic transduction	9.9 copies/μL	10^0^–10^6^ copies/μL	[50]
Thrombin/serum samples	Nanocarrier transduction	0.04 U/mL	0–0.8 U/mL	[51]
Anti-digoxin antibody, thrombin, and anti-HCV antibody/human serum	Nanocarrier transduction	26.1 pM for digoxin antibody, 78.3 pM for thrombin, 61.6 pM for HCV-Ab	0–5 nM for digoxin antibody, 0–5 nM for thrombin, 0–5 nM for HCV-Ab	[52]
*Candida albicans*/urine	Enzymatic transduction	/	/	[53]
SARS-CoV-2/throat swab samples	Nanocarrier transduction	10 copies/μL	10–10^4^ copies/μL	[54]
T4 polynucleotide kinase/human serum	Nanocarrier transduction	0.01 U/mL	0.01–0.5 U/mL	[55]
Human Epidermal Growth Factor Receptor 2/human serum	Nanocarrier transduction	0.6 pg/mL	1.0–1000.0 pg/mL	[56]
Hydrogen peroxide/tissue specimens	Nanocarrier transduction	0.1 μM	0.5–50 μM	[57]
MicroRNA-21/cell samples	Nanocarrier transduction	3.3 aM	10 fM–10 nM	[58]
DNA/serum sample	Nanocarrier transduction	4.02 pM	0.1–1000 nM	[59]
*Staphylococcus Aureus*	Nanocarrier transduction	67 cfu/mL	10^2^−10^6^ cfu/mL	[60]
The nucleocapsid phosphoprotein gene of SARS CoV-2/fetal bovine serum	Nanocarrier transduction	98 pM	0.1–20 nM	[61]
Prostate-specific antigen	Nanocarrier transduction	10 fg/mL	10 fg/mL–0.10 g/mL	[62]
Amyloid β oligomer/human serum and artificial cerebrospinal fluid samples	Nanocarrier transduction	0.22 pM	1 pM–250 pM	[63]
MicroRNA-21/serum sample	Nanocarrier transduction	7 pM	0–1 nM	[64]
*Staphylococcus aureus*/serum sample	Nanocarrier transduction	4.36 fM	/	[65]
MicroRNA-21/serum sample	Nanocarrier transduction	2.54 fM	/	[66]
MicroRNA/peripheral blood of rats	Nanocarrier transduction	329 aM	1 fM–100 pM	[67]
MicroRNA-21/serum sample	Nanocarrier transduction	5 pM	25–3000 pM	[68]
Carcinoembryonic antigen and human alpha fetoprotein antigen/human serum samples	Nanocarrier transduction	41.55 fg/mL for carcinoembryonic antigen; 14.28 fg/mL for alpha fetoprotein antigen	100.0 fg/mL–10.0 ng/mL for carcinoembryonic antigen; 100.0 fg/mL–10.0 ng/mL for alpha fetoprotein antigen	[69]
Rabies virus RNA/mouse brain tissue and muscle tissue samples	Nanocarrier transduction	6.3 copies/μL	/	[70]
Human immunodeficiency virus gene fragment/human serum and Hela cell lysate	Nanocarrier transduction	0.46 pM	2.5–75 pM	[71]
Cadmium, lead, and zinc	Enzymatic transduction	/	/	[72]
Exosomes	Nanocarrier transduction	54 particles/L	80–1.00 × 10^6^ particles/L	[73]
MicroRNA (let-7a)/serum sample	Nanocarrier transduction	48 pM	0.05–100 nM	[74]

**Table 2:** Applications of PGMs in non-glucose target quantitative food and environmental analysis.

Analytes/Real Sample	Signal Transduction Strategies	Limit of Detection	Linear Range	Ref.
Alkaline phosphatase/Fresh milk	Enzymatic transduction	0.13 U/μL	0.33–3.33 U/μL	[16]
*Staphylococcus aureus*/Peach juice, milk and water samples	Enzymatic transduction	2 cfu/mL	3–3 × 10^3^ cfu/mL	[78]
Norfloxacin/Animal-derived foods	Nanocarrier transduction	0.5 ng/mL	0.5–500 ng/mL	[79]
*Escherichia coli* O157:H7/Milk sample	Nanocarrier transduction	10 cfu/mL	10–10^7^ cfu/mL	[80]
*Salmonella*/Milk sample	Nanocarrier transduction	5 cfu/reaction	1–1 × 10^3^ cfu/reaction	[4]
Ampicillin/Milk sample	Nanocarrier transduction	2.5 × 10^−10^ mol/L	2.5 × 10^−10^–1.0 × 10^−7^ mol/L	[81]
*Escherichia coli* O157:H7/Milk sample	Nanocarrier transduction	10 cfu/mL	10–10^7^ cfu/mL	[82]
Ochratoxin A/Red wine	Nanocarrier transduction	0.88 pg/mL	1 pg/mL–300 ng/mL	[83]
Hg^2+^/Tap water and lake water	Glucose consumption	3.69 nM	5–30 nM	[84]
Cu^2+^/Tap water	Nanocarrier transduction	10 nM	0.01–5 μM	[85]
Ag^+^	Enzymatic transduction	4.6 μM	5–70 μM	[86]
Bacteria genes	Nanocarrier transduction	less than 100 molecular copies	/	[87]
Patulin/apple juice and grape juice	Nanocarrier transduction	0.05 ng/mL	0.1–50 ng/mL	[88]
*Escherichia coli* and *Staphylococcus aureus*/tap water	Nanocarrier transduction	*Escherichia coli* (3 cfu/mL); *Staphylococcus aureus* (7.59 × 10^2^ cfu/mL)	*Escherichia coli* (1.00 × 10^2^–1.00 × 10^7^ cfu/mL); *Staphylococcus aureus* (1.00 × 10^3^–1.00 × 10^7^ cfu/mL)	[89]

## Data Availability

Not applicable.
